# HCV-induced autophagy and innate immunity

**DOI:** 10.3389/fimmu.2024.1305157

**Published:** 2024-02-02

**Authors:** Jiyoung Lee, J.-H. James Ou

**Affiliations:** Department of Molecular Microbiology and Immunology, University of Southern California, Keck School of Medicine, Los Angeles, CA, United States

**Keywords:** HCV, autophagy, mitophagy, interferons, STING, inflammasome, oxidative stress

## Abstract

The interplay between autophagy and host innate immunity has been of great interest. Hepatitis C virus (HCV) impedes signaling pathways initiated by pattern-recognition receptors (PRRs) that recognize pathogens-associated molecular patterns (PAMPs). Autophagy, a cellular catabolic process, delivers damaged organelles and protein aggregates to lysosomes for degradation and recycling. Autophagy is also an innate immune response of cells to trap pathogens in membrane vesicles for removal. However, HCV controls the autophagic pathway and uses autophagic membranes to enhance its replication. Mitophagy, a selective autophagy targeting mitochondria, alters the dynamics and metabolism of mitochondria, which play important roles in host antiviral responses. HCV also alters mitochondrial dynamics and promotes mitophagy to prevent premature cell death and attenuate the interferon (IFN) response. In addition, the dysregulation of the inflammasomal response by HCV leads to IFN resistance and immune tolerance. These immune evasion properties of HCV allow HCV to successfully replicate and persist in its host cells. In this article, we discuss HCV-induced autophagy/mitophagy and its associated immunological responses and provide a review of our current understanding of how these processes are regulated in HCV-infected cells.

## Introduction

Despite significant advances in its treatment and the availability of efficacious direct-acting antiviral drugs (DAAs), hepatitis C virus (HCV) remains prevalent around the world with approximately 58 million chronic HCV carriers and 1.5 million new infections every year. Chronic HCV infection can lead to severe liver diseases including steatosis, cirrhosis and hepatocellular carcinoma (HCC). HCV is a positive-stranded RNA virus with a genome size of approximately 9600 nucleotides. Its RNA genome is packaged in an icosahedral capsid, which is further surrounded by a lipid envelope (1). HCV infection of hepatocytes is initiated by receptor-mediated endocytosis followed by the fusion of the viral envelope with the endocytic membrane and the release of its RNA genome into the cytosol. The RNA genome of HCV then directs the synthesis of viral proteins using the internal ribosome entry site (IRES) located near its 5’-end, producing a polyprotein that is roughly 3000 amino acids in length. Viral and host proteases proteolytically cleave the HCV polyprotein, giving rise to ten viral proteins, which are the core protein, E1 and E2 envelope proteins, the p7 viroporin, and nonstructural proteins NS2, NS3, NS4A, NS4B, NS5A and NS5B.

Although cells in response to HCV infection can generate antiviral responses, HCV has developed means to evade these antiviral responses to establish persistent infection. Approximately 75-85% of patients infected by HCV fail to clear the virus and become chronically infected. Autophagy can remove intracellular microbial pathogens and is a part of the antiviral responses. However, HCV exploits autophagy to overcome this host antiviral response. In this article, we will discuss the interaction between HCV-induced autophagy and the host innate immune responses. We hope this article will provide not only the information for understanding the HCV-host interaction to assist the development of HCV vaccines, which are not yet available, but also the information for understanding how cells may respond to other viral infections.

## Evasion of host innate immune response by HCV

### Pattern-recognition receptors (PRRs)

PRRs play important roles in triggering anti-microbial responses. They recognize pathogen-associated molecular patterns (PAMPs) and, upon binding to PAMPs, will trigger anti-microbial responses. There are three classes of PRRs, which are the retinoic acid-inducible gene-I (RIG-I)-like receptors (RLRs), toll-like receptors (TLRs) and nucleotide-binding oligomerization domain (NOD)-like receptors (NLRs). RLRs, including RIG-I, melanoma differentiation antigen 5 (MDA5) and the laboratory of genetics and physiology 2 (LGP2), are RNA helicases that recognize RNA PAMPs (2). RIG-I and MDA5 recognize double-stranded RNA (dsRNA). Although the specific PAMPs that they detect are different, their activation by HCV RNA can both lead to the production of interferons (IFNs) (3, 4). RIG-I recognizes the 5’-PPP and the 3’ poly U/UC sequence of the HCV genomic RNA (5). Once activated, the caspase activation and recruitment domain (CARD) of RIG-I interacts with the mitochondrial adaptor protein MAVS (also known as IPS-1, VISA or Cardif) on mitochondrial outer membranes. MAVS then further recruits TRAF3 and TRAF6 and subsequently activates the downstream TANK-binding kinase 1 (TBK1). TBK1 phosphorylates and activates Interferon Regulatory Factor 3 (IRF3), whose homodimerization and translocation to the nucleus activates the IFN genes. LGP2, which lacks the CARD domain, binds to the foreign RNA and synergizes with MDA5 to induce innate immune signaling (2).

### HCV proteins and their effects on interferon signaling pathways

**Figure 1:**
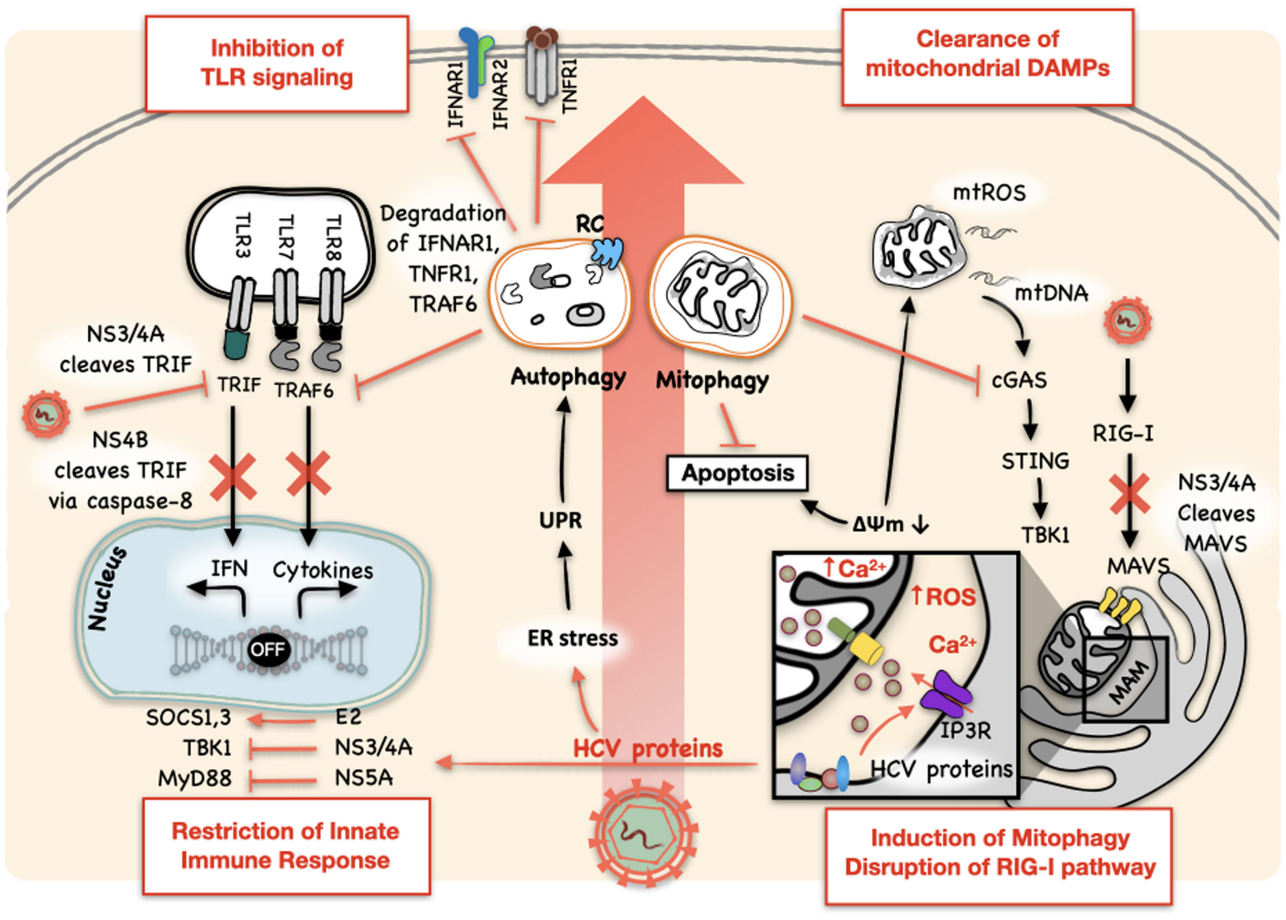
HCV inhibits the innate immune response. HCV proteins can directly inhibit the innate immune response. NS3/4A plays multiple roles in blocking the host innate immune response. It cleaves MAVS on MAM to block the RIG-I signaling pathway and TRIF to inhibit the TLR3 signaling pathway. NS4B can also disrupt TLR3-mediated IFN signaling by inducing the degradation of TRIF via caspase-8. Other HCV proteins had also been shown to regulate and restrict the production of IFNs and their antiviral signaling: E2 induces SOCS1 and SOCS3; NS3/4A sequesters STING to inhibit the activation of TBK1; NS5A sequesters MyD88 to inhibit TLR signaling; and NS5B induces and activates PKR to inhibit the expression of IFNs and ISGs. HCV also usurps the antiviral activity of autophagy and uses it for its own replication. It not only utilizes the autophagic membranes as the sites for its RNA replication and the production of infectious HCV particles, but also manipulates autophagy to regulate type I IFN response by promoting the autophagic degradation of IFNAR1, TNFR1, and TRAF6, important signal transducers for the activation of NF-κB and the expression of pro-inflammatory cytokines. HCV-induced mitophagy also regulates innate immune response by clearing mitochondrial DAMPs, namely mtROS and mtDNA, and damaged mitochondria themselves, which otherwise would activate the cGAS/STING/TBK1 pathway to induce the expression of IFNs. Mitophagy also inhibits premature apoptotic cell death and promotes HCV persistence. RC: HCV RNA replication complex. The mitochondria-associated membrane (MAM) is boxed and enlarged in the inset to reveal the Ca^2+^ transporter IP3R.

Hepatocytes infected by HCV produce type I and type III IFNs and upregulate the expression of IFN-stimulated genes (ISGs). Type I IFN-α and IFN-β bind to the heterodimer of IFN-α receptor (IFNAR) 1 and 2, type II IFN-γ binds to the heterodimeric complex of IFN-γ receptor (IFNGR) 1 and 2 and type III IFN-λ binds to the heterodimeric complex of IFN-λ receptor (IFNLR) and IL-10Rβ. Type I and III IFN signaling is mediated by Janus kinases (JAKs) and STAT proteins and induces the expression of ISGs including ISG15, 2’,5’-oligoA synthase (OAS), protein kinase RNA-activated (PKR), the GTPase myxovirus resistance 1 (Mx1) and ribonuclease L (RNase L), to name a few (6). Nonetheless, HCV proteins can dampen the host IFN response to enhance its replication (**Figure 1**). NS3/4A, the viral serine protease complex of HCV, plays a crucial role in hampering the antiviral signaling of IFNs. NS3/4A cleaves MAVS, the adaptor protein of the RIG-I signaling (7, 8). The introduction of a point mutation at cysteine-508 (Cys-508) to prevent MAVS from being cleaved by NS3/4A restored the IFN production in HCV-infected cells or cells containing an HCV subgenomic RNA replicon (4, 9). The mitochondria-associated membranes (MAMs) at the endoplasmic reticulum (ER)-mitochondria contact site serve as the site for MAVS signaling (**Figure 1**) (8). Also, NS3/4A can cleave peroxisome-associated MAVS and disrupt RIG-I signaling initiated from peroxisomes (10). In addition, NS3/4A can cleave the TIR-domain containing adapter-inducing IFN-β (TRIF), an adaptor protein of the TLR3 signaling pathway (**Figure 1**) (10, 11). HCV protein NS4B was also reported to induce the degradation of TRIF via caspase-8 (12). Other HCV proteins like core, E2, NS4B, and NS5A had also been shown to be involved in the resistance to IFNs by inducing the suppressor of the cytokine signaling proteins 1 and 3 (SOCS1 and 3), sequestering STING to inhibit the activation of TBK1 and sequestering MyD88 to inhibit TLR signaling [for more details, see reviews (13–15)]. In 293T cells, NS3/4A bound to TBK1 and interfered with its association with IRF3 (16). PKR, a dsRNA-activated kinase, is an antiviral protein that is also induced by IFNs. HCV NS5B has been shown to induce the activation of PKR and the phosphorylation and inactivation of its downstream effector eIF2α, a protein translation initiation factor, to suppress the production of IFNs, ISGs and MHC class I (**Figure 1**) (17–20).

## HCV-induced autophagy and innate immune response

### HCV-induced autophagy

Autophagy is a catabolic process by which cells deliver damaged organelles and dysfunctional cellular components to lysosomes for degradation. In the early stage of autophagy, a crescent membrane structure termed phagophore appears in the cytoplasm. The subsequent expansion of the membranes of phagophores leads to the formation of an enclosed double-membrane structure termed autophagosomes. Autophagosomes fuse with lysosomes to form autolysosomes. The cargoes of autophagosomes are then digested by lysosomal enzymes in autolysosomes for recycling. Autophagy is important for maintaining cellular homeostasis. It is closely associated with the growth and energy state of the cell, and the activation of mTOR suppresses the initiation of autophagy. mTOR regulates cell growth and metabolism, integrates the cellular nutrient state with environmental signals and controls the initiation of autophagy by regulating the activation of AMPK, the AMP sensor. HCV induces autophagy via multiple pathways, including the expression of viral proteins, nutrient exploitation, alteration of cellular metabolism and production of reactive oxygen species (ROS). Interaction of HCV NS3 with immunity-related GTPase family M protein (IRGM) is sufficient to induce autophagy (21). HCV triggers the activation of both IRGM and early autophagy initiator Unc-51-like kinase 1 (ULK1), which are required for membrane remodeling and Golgi fragmentation, to initiate autophagy (22). NS4B can also bind to Beclin-1, hVps34 and Rab5 to initiate autophagy [for a detailed review, see (23)].

As mentioned above, intracellular events like the ER stress and the generation of ROS from mitochondria in response to HCV infection can also induce autophagy. HCV-induced ER stress stimulates the unfolded protein response (UPR), which is triggered by the activation of the inositol-requiring enzyme 1 (IRE1), the activating transcription factor 6 (ATF6) and the double-stranded RNA-activated protein kinase-like ER kinase (PERK) (24). The UPR attenuates protein synthesis, promotes chaperone-mediated protein folding and facilitates the degradation of misfolded proteins to alleviate the ER stress (23). The silencing of IRE1, ATF6 or PERK to disrupt the UPR, which also disrupts HCV-induced autophagy, or the silencing of autophagy-related proteins Atg5, Atg7 and Atg12 suppressed the replication of HCV, indicating that the UPR and autophagy benefit HCV replication (24–27) (**Figure 1**). The importance of autophagy in HCV infection can be partially attributed to the fact that autophagic membranes, where lipid rafts rich in cholesterol and sphingolipids are also localized, can serve as the sites for the assembly of the HCV RNA replication complex (24, 28–30). ROS is a byproduct of mitochondrial aerobic respiration. The enhanced generation of ROS or oxidative stress was observed in cell cultures infected by HCV and in the liver of chronic hepatitis C patients (31–33). The elevation of ROS prompted the phosphorylation of the sequestosome protein p62 at Ser349, which is implicated in the initiation of autophagy (33). p62 binds to poly-ubiquitinated proteins for their sequestration in autophagosomes for autophagic degradation. Although ROS has antiviral potential as demonstrated in the studies of different viruses, HCV as well as human immunodeficiency virus type 1 (HIV-1), influenza virus and dengue virus exploits ROS-induced autophagy for their genome replication and immune suppression (33–35).

The term xenophagy depicts the process by which intracellular microbial pathogens are removed by autophagy. The removal of viruses by autophagy is termed virophagy (36, 37). These terms describe autophagy as a host antiviral response that clears intracellular viral particles (37). However, HCV can usurp the antiviral activity of autophagy and use it to benefit its own replication (24, 38) (**Figure 1**). First, HCV can use autophagic membranes as the sites for its RNA replication (24, 27, 30). Second, HCV induces Rubicon to delay the maturation of autophagosomes to maximize its RNA replication (24, 39) (see below). Third, HCV promotes the interaction between its envelope protein E2 and apolipoprotein E (ApoE), which is important for enhancing the infectivity of HCV, using autophagic membranes (29, 40). Fourth, autophagy suppresses type I IFN response (for detailed reviews, see (41, 42)), and the silencing of UPR- or autophagy-related genes increased the expression of ISGs and reduced HCV replication (25). Finally, HCV dampens the innate immune response by stimulating the autophagic degradation of TRAF6, an adaptor protein important for the activation of nuclear factor-κB (NF-κB) and the expression of pro-inflammatory cytokines (24, 34, 42), as well as the autophagic degradation of TNFR1 and IFNAR1, which are important for the IFN response (43, 44).

### HCV-induced autophagy and IFN response

**Figure 2:**
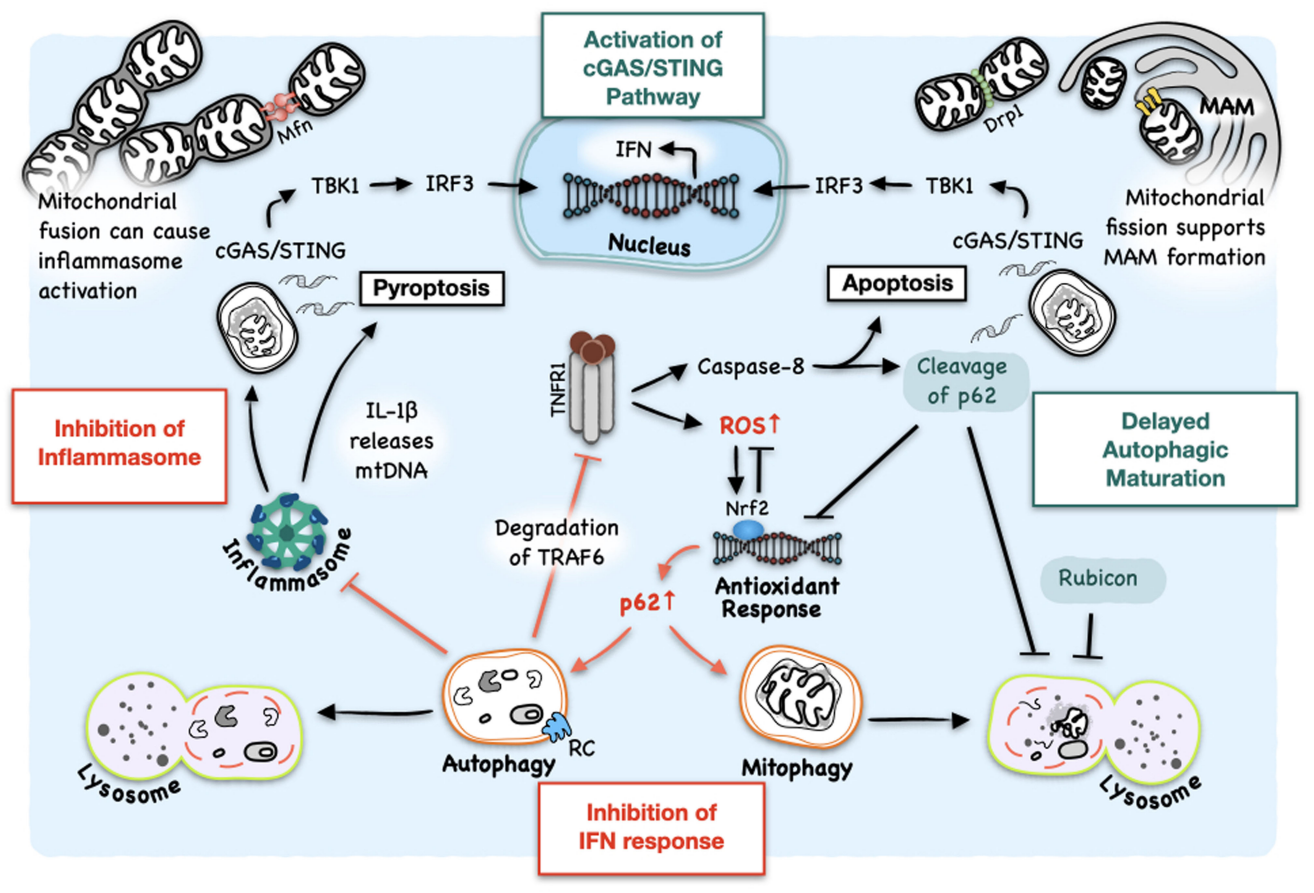
Interplay between HCV-induced autophagy and host innate immune response. HCV-induced autophagy removes DAMPs to inhibit the formation of inflammasomes and hence pyroptosis. HCV-induced autophagic degradation of TNFR1 also inhibits the activation of caspase-8 and apoptosis. However, host cells in response to HCV infection also express TNF-α and IFNAR2 (also see **Figure 1**). TNF-α at a low concentration causes the generation of ROS to initiate autophagy. However, it, at a high concentration, can also put a halt on the completion of autophagy via caspase-8-mediated cleavage of p62, ultimately activating the cGAS/STING/TBK1 pathway and apoptosis. TNF-α-induced cleavage of p62 also disrupts Nrf2 signaling, which is activated by HCV to induce the expression of antioxidant genes to alleviate oxidative stress. HCV also delays the maturation of autophagosomes by inducing the expression of Rubicon for the accumulation of autophagosomes to support its RNA replication, which can also lead to the accumulation of DAMPs. Mitochondrial dynamics are closely related to the regulation of innate immune response. On the other hand, mitochondrial fusion supports inflammasome activation and pro-survival pathways that, if prolonged, can contribute to tumorigenesis. On the other hand, mitochondrial fission, which is coupled with mitophagy and promoted by HCV, supports the formation of MAM which can serve as the platform to mediate IFN response. RC: HCV replication complex.

The interplay between HCV-induced autophagy and the host immune response has attracted a lot of attention (45, 46). Under normal conditions, cells maintain a basal autophagy activity to regulate energy and nutrient states and remove damaged organelles and protein aggregates to maintain cellular homeostasis. Numerous disorders like heart diseases, neurodegenerative diseases and tumors are associated with the impairment of autophagy, which leads to disruption of many intracellular processes. Among them is innate immune responses (47). Autophagy also regulates innate immune responses in HCV-infected cells. Although there were conflicting results regarding whether the inhibition of autophagic initiation affected the type 1 IFN pathway in HCV-infected cells (25, 46), the silencing of RUN Domain Beclin-1-interacting and cysteine-rich domain-containing protein (Rubicon) stimulated the IFN response. Rubicon plays a critical role in the control of maturation of autophagosomes (48, 49). It can bind to the complex of UV radiation resistance-associated gene (UVRAG) and class III phosphatidylinositol-3-kinase (PI3KC3) to suppress the maturation of autophagosomes (48). It can also sequester UVRAG from class C vacuolar protein sorting (C-Vps)-homotypic fusion and protein sorting (HOPS) complex to inhibit the activation of Rab7 and the fusion between autophagosomes and lysosomes (49). HCV induces the expression of Rubicon in the early stage of infection to prevent the maturation of autophagosomes, leading to the accumulation of autophagosomes to enhance HCV RNA replication (39, 46). However, the inhibition of this late-stage of autophagy, which prevents the autophagic protein degradation, also activates the innate immune response (46) (**Figure 2**). The silencing of autophagic proteins like LC3B to prevent the formation of autophagosomes or the treatment with chloroquine to inhibit autophagic protein degradation enhances IFN-β production and IFN signaling in HCV-infected cells (25). In addition, Chandra et al. showed that IFNAR1 was degraded by autophagy induced by HCV infection (43). The use of thapsigargin (TG) to induce ER stress or the inhibition of mTOR downregulated IFNAR1, and the inhibition of autophagy by silencing Atg7 or PERK upregulated the expression of IFNAR1 in the presence of HCV. As a result, the alleviation of the ER stress or the inhibition of autophagy using chemical inhibitors or siRNA enhanced viral clearance in cell cultures treated with both IFN-α and ribavirin (RBV) (43). These results demonstrated an important role of autophagic protein degradation, the late stage of autophagy, in the control of IFN response in HCV-infected cells. In contrast to HCV, it was shown that the silencing of Rubicon with shRNA, which promoted the maturation of autophagosomes, enhanced IFN-β and IL-6 production and inhibited the replication of H1N1 influenza virus and vesicular stomatitis virus (VSV) (50).

### HCV-induced autophagy and antioxidant response

NF-E2-related factor 2 (Nrf2) activates antioxidant gene expression in response to oxidative stress. The E3 ubiquitin ligase Kelch-like ECH-associated protein 1 (Keap1) associates with Nrf2 to keep it in an inactive state. The killer inhibitory receptor (KIR) domain of p62 allows p62 to bind to Keap1 to free Nrf2. This allows Nrf2 to move into the nucleus to bind to the antioxidant response element (ARE) to induce the expression of antioxidant genes (51). Since the p62 promoter contains the ARE, the activated Nrf2, in turn, induces the expression of p62 (52). On the other way around, Keap1, by binding to p62, prevents p62 from interacting with LC3 and delivering cargoes to autophagosomes for autophagic degradation. This can impede the autophagic removal of dysfunctional mitochondria under oxidative stress and activate the cGAS/STING pathway to lead to the production of IFN (more details below). In the meantime, p62 can associate with Raptor to activate mTOR complex 1 (mTORC1) and promote the pro-survival pathway (53). The activation of Nrf2 in response to HCV infection is mediated by the mitogen-activated protein kinase (MAPK) p38 and JAK, and the knockdown of Nrf2 inhibits the phosphorylation of the kinase Akt and its downstream effector Bad, suggesting a role of Nrf2 in the survival of HCV-infected cells and their possible oncogenic transformation (54). Indeed, the accumulation of p62 was observed in multiple cancers including tumor tissues positive for HCV (55, 56).

### HCV-induced autophagy and inflammasome

Inflammasome is a multiprotein complex composed of an NLR such as NLRP3, the adaptor protein apoptosis-associated speck-like protein containing a CARD (ASC) and the effector protease caspase-1. It can be activated by PAMPs, damage-associated molecular patterns (DAMPs), and toxins (57). The assembly of inflammasomes leads to the activation of caspase-1, which then cleaves IL-1β and IL-18 to produce their mature form for release from cells. The formation of inflammasomes also triggers pyroptosis, a regulated form of cell death associated with a high inflammation state. Caspase-1 also cleaves Gasdermin D (GSDMD) to produce GSDMD-C and GSDMD-N. GSDMD-N oligomerizes to form membrane pores on the plasma membrane for the release of IL-1β and IL-18 and eventually leads to pyroptosis (57, 58).

In response to HCV infection, the NLRP3 inflammasome is activated in hepatocytes and macrophages, leading to the release of IL-1β (59, 60). Elevated serum level of IL-1β was observed in chronic HCV patients as well (61). HCV can also enter Kupffer cells, the resident macrophages of the liver, and trigger the TLR7-mediated inflammasome activation and the release of IL-1β (61). The expression of HCV proteins, the increase of ROS, the induction of ER stress and other organelle stresses caused by HCV infection may exacerbate the inflammasome response (59). Lipid accumulation was shown to activate the NLRP3 inflammasome as well (59, 62, 63). The HCV core protein can also stimulate IL-1β production from macrophages, and this process requires HCV core-induced Ca^2+^ mobilization and phospholipase C activation (59, 61).

Autophagy, via lysosomal degradation, eliminates the activators of inflammasomes, such as inflammasome components, cytokines, mitochondrial ROS (mtROS), mitochondrial DNA (mtDNA) and damaged mitochondria. Thus, autophagy negatively regulates the NLRP3 inflammasome to prevent excessive inflammation, which can lead to the development of cancer (64). HCV induces the expression of Rubicon to inhibit the maturation of autophagosomes and disrupt the autophagic flux at as early as 6 hours post-infection in cell cultures (39). It is conceivable that this disruption of autophagic flux plays a role in the induction of inflammasomes and pyroptosis, which are detected in cells 2-3 days after HCV infection (60). HCV also induces the expression of NLRP3, an important component of inflammasomes, and the silencing of NLRP3 inhibits HCV-induced Golgi fragmentation, which is a possible source of autophagosomal membranes in HCV-infected cells (65). Thus, there appears to be a two-way crosstalk between autophagy and inflammasomes in HCV-infected cells.

### HCV-induced inflammasome and innate immune response

The prompt removal of cytotoxic agents by autophagy protects cells from dangers, and the insufficient induction of autophagy or delay in autophagic degradation can cause hyperinflammatory responses (66). HCV delays the maturation of autophagosomes for autophagosomal membranes to accumulate to support the replication of its genome. However, the inflammation may arise due to the delay in the autophagic clearing of DAMPs. Aarreberg et al. found that IL-1β could induce the release of mtDNA into the cytosol to activate cyclic GMP-AMP synthase (cGAS), a cytosolic DNA sensor (**Figure 2**). This led to the production of the second messenger cyclic GMP-AMP (cGAMP) and the activation of STING followed by the activation of IRF3 and NF-κB to induce the expression of IFNs and proinflammatory cytokines (67). Type I IFNs, in turn, can repress the NLRP1- and NLRP3-dependent IL-1β production via the STAT1 transcription factor, which can also induce the expression of IL-10 to activate STAT3 through an autocrine mechanism to suppress the expression of pro-IL-1α and pro-IL-1β (68). Nitric Oxide (NO) induction or inducible nitric oxide synthase (iNOS) deficiency induced by TNF-α or IFN-γ suppresses inflammasome activation, preventing prolonged inflammation (69, 70). The mutually exclusive relationship between inflammasome and IFN production is well depicted by Banerjee et al. (71). They showed that inflammasome- and caspase-1-mediated cleavage of GSDMD and the subsequent formation of GSDMD pores led to K+ efflux and negatively regulated the IFN-β production, and GSDMD knockout enhanced the activation of TBK1 and IRF3 in response to intracellular poly(dA:dT) or *Francisella novicida* infection (71). In HCV-infected cells, Wallace et al. observed that pyroptosis preceded apoptosis, and the depletion of NLRP3 increased the activation of caspase-3, an executioner caspase of apoptosis (60) (**Figure 2**), most likely due to the increase in innate immune signaling (72). Interestingly, caspase-3 knockout also decreased the activation of caspase-1, suggesting a possible role of caspase-3 in the initiation of pyroptosis (60). The continuous interplays between IL-1β and IFN in the microenvironment of HCV-infected cells may contribute to immune tolerance, IFN resistance, an extended inflammation state and the development of liver cirrhosis and HCC (73, 74) (**Figure 2**). This may also be the reason why the interferon therapy does not generate sustained virological response in the great majority of HCV patients (75).

## HCV-induced mitophagy and innate immune response

### HCV-induced mitophagy and immune response

Maintaining a healthy pool of mitochondria is critical for cellular function and survival. Mitochondria undergo constant fusion and fission via guanosine triphosphatases (GTPases)-dependent activities. Mitofusins (Mfn1 and Mfn2), fusogenic transmembrane GTPases located on the mitochondrial outer membrane, bridge two adjacent mitochondria to promote mitochondrial fusion. Dynamin-related protein 1 (Drp1) and its receptor mitochondrial fission factor (Mff) promote mitochondrial fission by forming helical oligomers of Drp1 that encircle the mitochondrial outer membrane. Healthy mitochondria maintain a low Ca^2+^ level in their matrix where matrix dehydrogenases and oxidative phosphorylation produce ATP. Mitochondrial dysfunction can cause a reduction in the mitochondrial membrane potential (ΔΨm) due to uncontrolled production of ROS or the mitochondrial Ca^2+^ influx. The decrease in ΔΨm leads to the opening of the mitochondrial permeability transition pore (MPTP), through which mtDNA and cytochrome c are released (76). Dysfunctional mitochondria and the accumulation of DAMPs eventually lead to the disruption of ATP production and promote anaerobic glycolysis (77). Ca^2+^ overload can also lead to mitochondrial fragmentation, the disruption of ATP synthesis, and apoptosis (78, 79).

Mitochondria-associated membranes (MAMs), the contact sites of ER and mitochondria, create microdomains with high flux of Ca^2+^ where the transportation of Ca^2+^ occurs (**Figure 1**). Ca^2+^ goes through ER-resident calcium transporters (RyR/IP3R) and mitochondrial voltage-dependent anion channel type 1 (VDAC 1) on the outer membrane of mitochondria (OMM) and the calcium uniporter (MCU) on the inner membrane of mitochondria (IMM). Mitochondrial fusion leads to the loss of MAMs where MAVS relays the RIG-I signaling, and failure in maintaining MAMs can compromise innate immunity. Instead, the fusion of dysfunctional mitochondria can lead to the activation of inflammasome (80) via the accumulation of mtROS and mtDNA within the prolonged network of damaged mitochondria (77). Ichinohe et al. showed that endogenous NLRP3 was associated with Mfns after the infection of influenza virus or encephalomyocarditis virus (EMCV) in the LPS-primed bone marrow-derived macrophages (BMMs) (80). The treatment with carbonyl cyanide m-chlorophenyl hydrazone (CCCP), a protonophore that dissipates ΔΨm, or the knockdown of Mfn2 decreased the secretion of IL-1β (80).

Mitochondrial fission precedes mitophagy, a selective autophagy that removes damaged mitochondria for degradation (77). Failure in clearance of damaged mitochondria will lead to the accumulation of DAMPs, particularly mtDNA, to trigger the transcription and translation of inflammatory cytokines (77). The immune-evoking signals like cytochrome c, ROS and mtDNA from damaged mitochondria can also stimulate host responses. During HCV infection, MAMs can provide a site for the replication of the viral genome (81). HCV proteins present in the MAMs cause mitochondrial Ca^2+^ overload followed by local production of ROS and trigger the collapse of ΔΨm that leads to apoptosis, eliminating HCV-infected cells (82). Although it was noted that HCV-induced ROS can play a role in restricting the replication of HCV (83–85), mitochondrial fission and mitophagy induced by HCV infection in cell cultures or in patients or by the expression of HCV protein NS5A alone can overcome the antiviral effect of ROS and ROS-induced apoptosis (86–89). By removing damaged mitochondria along with mitochondrial DAMPs, mitophagy plays a critical role in preventing cell death caused by damaged mitochondria and mtROS. Evidence showed that HCV could upregulate mitophagy-related proteins including Drp1, the Drp1 receptor Mff, Parkin and PINK1 (86, 87), and the inhibition of PINK1 or Parkin decreased HCV replication, suggesting a positive role of mitophagy in HCV infection (86). PINK1 is a protein kinase that phosphorylates and recruits Parkin, an E3 ubiquitin ligase, to the outer membrane of mitochondria. Parkin can then ubiquitinate mitochondrial outer membrane proteins to trigger mitophagy.

MAMs also serve as the sites where immune signaling complexes form to control the viral infection. In the case of influenza virus, the fusion state of mitochondria in A549 cells, a human lung cancer cell line, was associated with the translocation of Drp1 from the mitochondria to the cytosol after viral infection (90). MAMs restored after the fragmentation of mitochondria by Mito-C, a pro-fission compound, increased IFN production for viral clearance (90). Similarly, the silencing of MFN2 enhanced the IFN-β promoter activity and reduced the permissiveness of cells to HCV infection (8). It is of note that the depletion of Drp1 suppressed HCV release with an increase in innate immune response and apoptosis (87).

The antagonistic relationship between mitophagy and apoptosis in the context of immune response was recently confirmed by Kim et al. who found that HCV-induced mitochondrial fission coincided with the attenuation of apoptosis (87). The authors added that the increase in caspase-3 activation and mitochondria-mediated apoptosis was linked to the increase in innate immune signaling (87). The pro-survival and proliferation nature of mitophagy was established as Drp1-mediated mitochondrial fission was shown to deplete the tumor suppressor p53 and increase the phosphorylation of the retinoblastoma (Rb) protein, resulting in the G1/S cell cycle progression and tumorigenesis (91–93). Plus, under the hypoxic condition, nuclear p53 represses the expression of Bcl-2 interacting protein 3 (BNIP3) thereby attenuating mitophagy (94). BNIP3 induces mitophagy by triggering mitochondrial membrane depolarization (94).

Intriguingly, evidence showed that the delay in autophagy could prime cells for apoptosis. In CD14+ cells, IFN-α impaired autophagic degradation of mitochondria, resulting in the accumulation of mtDNA and ultimately the STING-mediated immune response (95). In HCV-infected cells, the inhibition of autophagosomal maturation by the upregulation of Rubicon also resulted in the accumulation of damaged mitochondria (39), which then activated the IFN pathway (46), and led to both apoptosis and pyroptosis (96).

### TNF alpha-induced mitophagy and innate immune response

TLR7 and TLR8 can sense HCV RNA in the endosome and induce the expression of tumor necrosis factor-α (TNF-α) to suppress HCV replication (44, 97). TNF-α signaling can activate NF-κB and IRFs, leading to the production of pro-inflammatory cytokines and IFNs, respectively. TNF-α induced by HCV is necessary for type I IFN signaling in HCV-infected cells, as the silencing of TNF-α or its receptor TNFR1 abrogates the expression of IFNAR2 and desensitizes HCV-infected cells to type I IFNs (44, 98).

TNFR1 exists in two temporally and spatially distinct signaling complexes: TNFR1 complex I (TNFR1-CI) and II (TNFR1-CII) (99, 100). TNFR1-CI, formed on the plasma membrane, recruits RIPK1, TRADD, TRAF2 and the cellular inhibitor of apoptosis protein (cIAP) 1 and 2. cIAP proteins add K63, K11 and K48 poly-ubiquitin chains to themselves and RIPK1. This further recruits linear ubiquitin chain assembly complex (LUBAC) to add M1 linear polyubiquitin chain to RIPK1 and fully activates TNFR1-CI and NF-kB. TNFR1-CII is formed when RIPK1 becomes deubiquitinated and released from TNFR1-CI. TNFR1-CII, assembled after the internalization of TNFR1, recruits FADD, caspase-8 and FLICE-like inhibitory protein (FLIP). The degradation of cIAPs and the activation of caspase-8 initiate the apoptotic pathway. In the case of caspase inhibition, RIPK3 associates with RIPK1 to induce necroptosis. Zhao et al. reported that the cleavage of p62 by caspase-8, a key protease in extrinsic apoptotic signaling initiated from death receptors including TNFR1, prevented p62 from participating in autophagy (101) (**Figure 2**). The mutation of D329, the caspase-8 cleavage site in p62, to histidine (D329H) or glycine (D329G) rendered p62 resistant to caspase-8 and inhibited caspase-8-induced apoptosis. This cleaved p62 lacks the KIR domain and can no longer bind to Keap1, which associates with Nrf2 to inhibit its activity (101). Thus, the activity of caspase-8 on p62 shuts off the oxidative stress response and at the same time inhibits further expression of p62 and autophagic degradation. As mentioned above, the association of Keap1 with p62 attenuates autophagic degradation and in the meantime activates Nrf2-mediated oxidative stress response (51). An interesting biphasic effect of TNF-α on the Keap1/Nrf2 pathway had been observed, with TNF-α activating and promoting the nuclear localization of Nrf2 at low concentrations and impairing the antioxidant signaling of Nrf2 to result in severe oxidative stress and cell death at high concentrations (102). The activation of Nrf2 in response to HCV was mediated by the p38 MAPK and JAK, and the silencing of Nrf2 inhibited the phosphorylation of Akt and its downstream effector Bad, implicating Nrf2 in the survival of HCV-infected cells and their potential oncogenic transformation (54). As discussed above, the inhibition of autophagic degradation of mitochondria under oxidative stress can activate the cGAS/STING pathway to induce the expression of IFNs. Oroxylin A is a flavonoid compound isolated from *Scutellaria baicalensis*. It has antioxidant, anti-inflammatory and anti-tumor activities (101). The apoptosis of liver tumor cells induced by oroxylin A is dependent on p62-mediated activation of caspase-8, which cleaves p62 at D329 to remove its KIR domain (101). The cleavage of p62 by caspase-8 down-regulated Nrf2 and reduced the oxidative stress response (**Figure 2**). However, it is noteworthy that HCV fights the action of TNF-α and TNFR1 signaling by inducing the autophagic degradation of TNFR1 (44). It should be noted as well that TNF-α had also been shown, through a paracrine mechanism, to disrupt the tight junctions and promote the entry of HCV into polarized hepatocytes, hence promoting HCV infection (103). In Huh 7.5 cells, HCV-induced autophagic degradation of IFN-α receptor, IFNAR1 rendered the infected cells resistant to IFN treatment (43).

## Concluding remarks

Autophagy, a process used by the cell to maintain its homeostasis, also plays important roles in antiviral responses. Besides its role in virophagy, autophagy influences other innate immune pathways including PRR signaling, the formation of inflammasomes, and the expression of pro-inflammatory cytokines and IFNs. HCV-induced autophagy affects all these intracellular antiviral responses.

HCV induces autophagy/mitophagy to promote its replication. Mitophagy is a quality control mechanism used by the cell to maintain a healthy pool of mitochondria, which, if damaged, can trigger cell death. To cope with the HCV-associated damage in mitochondria, HCV induces mitophagy to remove dysfunctional mitochondria to prevent premature cell death. Rubicon is induced by HCV to delay the maturation of autophagosomes, causing the accumulation of autophagosomal membranes, which support the HCV genome replication (23). This delay in autophagic degradation can also actuate type I IFN signaling (46). In this regard, TNF-α-induced cleavage of p62 by caspase-8 may have the same effect as Rubicon in delaying autophagosome maturation and on IFN signaling.

Mitochondrial fission and fusion (i.e., mitochondrial dynamics) also affect HCV replication. Mitophagy is often coupled with mitochondrial fission. HCV-induced mitochondrial fission correlates with the attenuation of apoptosis (87). Interestingly, the inhibition of mitochondrial fission affected the secretion of progeny viruses (87), indicating a possible role of mitophagy in the release of infectious viral particles. The perturbation of mitochondrial metabolism can desensitize HCV-infected cells to IFN-α. These immune-antagonizing activities of HCV explain why most HCV patients fail to eradicate this virus and eventually develop severe liver diseases and why IFN therapies did not generate sustained response in the majority of patients infected by HCV.

In patients, HCV can induce inflammasomes to produce mature IL-1β, mainly by Kupffer cells. IL-1β induces the release of mtDNA that can activate cGAS/STING pathway to produce IFNs. This expression of IFNs in turn inhibits the formation of inflammasomes to repress further generation of the mature IL-1β. By inducing autophagic degradation of the components of inflammasomes, HCV hampers the inflammasome formation. This interplay between IL-1β and IFN likely plays an important role in HCV persistence and pathogenesis.

HCV-induced mitochondrial oxidative stress triggers Nrf2 activation. p62, an adaptor molecule important for autophagic/mitophagic protein degradation, binds to Keap1 to activate the Nrf2-mediated antioxidant response (56). The Nrf2 activation in turn promotes the expression of p62, thereby enforcing the antioxidant response and mitophagy, which helps to remove immune-evoking DAMPs and promote the survival of infected cells, potentially inducing tumorigenesis.

There is a constant battle between viruses and their host cells for survival. One such battleground is the control of the autophagic pathway. HCV has developed remarkable mechanisms to control this pathway not only to promote its replication but also to attenuate the host innate immune response. The successful suppression of expression of cytokines and IFNs and their signaling pathways plays a critical role for HCV to establish chronic infection in the great majority of patients that it infects. The prolonged perturbation of the autophagic pathway by HCV likely plays an important role in HCV pathogenesis. The current DAAs, which target HCV NS3 protease, NS5A and/or NS5B polymerase to inhibit HCV replication, can also alleviate the long-term effect of HCV on this important cellular pathway to restore the health of hepatocytes.

## Author contributions

JL: Writing – original draft, Writing – review & editing. J-HO: Writing – review & editing.
